
JOURNAL INFORMATION
==============================
NLM Title Abbreviation: Hist Cienc Saude Manguinhos
Journal ID: Hist Cienc Saude Manguinhos
NLM Journal ID: 9513999
Journal ID: hcsm

História, Ciências, Saúde - Manguinhos

ISSN: 0104-5970
EISSN: 1678-4758
Publisher: Casa de Oswaldo Cruz - Fiocruz

ARTICLE INFORMATION
==============================
PMCID: PMC10561976
DOI: 10.1590/S0104-59702023000100047
Article ID: 01511
Article version: 1
Subjects: Reseñas

Hospices, asylums and psychiatric institutions in Ibero-America

Hospicios, manicomios e instituciones psiquiátricos en Iberoamérica

http://orcid.org/0000-0003-4209-4909 López Manuela Barrios i
i Estudiante del doctorado en historia, Universidad de Guanajuato.Guanajuato – Guanajuato – México mbarrios@unal.edu.co
Electronic publication date: 2023 Oct 9
Publication date: 2023
Volume: 30
Electronic Location ID: e2023047
Product: RÍOS Andrés RUPERTHUZ Mariano org.  
De manicomios a instituciones psiquiátricas: experiencias en Iberoamérica, siglos XIX y XX.
Ciudad de México: Unam: Madrid: Sílex Ediciones S.2022. 640.
License: This is an Open Access article distributed under the terms of the Creative Commons Attribution License, which permits unrestricted use, distribution, and reproduction in any medium, provided the original work is properly cited.
License URL: https://creativecommons.org/licenses/by/4.0/

Figure count: 0
Table count: 0
Equation count: 0
Reference count: 4

==============================

Hacer del espacio un actor histórico supone una relocalización de conceptos clave en la historiografía de la locura desde nuevas miradas sobre el tiempo, el espacio y la historia misma. En este compilado de 13 artículos cada autor, desde una ciudad distinta del ámbito iberoamericano, ha hecho de los espacios manicomiales el punto medular de su escritura. El objetivo del libro ha quedado claramente delimitado en la introducción, al pretender relacionar el saber médico que se especializó en la locura con un proceso histórico de cambios en todos los niveles, con impacto en la vida cotidiana, en el arte, en la idea del individuo, en las ideas políticas: en la modernidad (Ríos, Ruperthuz, 2022, p.19). Entendida como un momento histórico donde los Estados nacionales se preocuparon por ejercer un control del espacio – no siempre de manera exitosa –, fue un periodo de acciones con impacto directo en la población que visibilizó los márgenes de las sociedades donde algunos individuos no encajaron.

El grupo de investigadores detrás de estos textos tiene una larga trayectoria en el estudio histórico de la locura que en el fondo no es más que la pregunta por aquello que la sociedad ha hecho con la diferencia, con lo inaprensible, con eso que se escapa del entendimiento de las mayorías. Paradójicamente, la modernidad como esa especie de aire en común que se respiraba en una época de transiciones, rupturas y nuevos referentes ideológicos fue simultáneamente un tiempo donde se amplió la brecha de marginalidad exponencialmente. Mientras las ciudades cambiaban sus referentes estéticos para vestirse de gala, otros cuerpos con sus comportamientos empezaron a encarnar una existencia por fuera de los límites naturalizados, como bien lo dijo la investigadora argentina Graciela Frigerio, “los cuerpos de la locura no son cuerpos bien perfumados” (30 oct. 2022). Las categorías clínicas del saber psiquiátrico para clasificar la locura dan cuenta de una diferenciación en función de los contextos iberoamericanos; cuerpos y comportamientos etiquetados con adjetivos que los calificaron como "esclavos", "liberados", "pobres", "trabajadores", "pensionistas", "pacíficos", "turbulentos", "furiosos", "sucios" o "epilépticos", los cuales ayudaron a separar los individuos internados en manicomios con criterios más morales o sociales y menos clínicos.

Por otra parte, es frecuente encontrar en la historiografía referente al encierro manicomial la conceptualización propuesta por Michel Foucault como estandarte de las investigaciones históricas sobre la locura, al homologar en las sociedades occidentales su formulación teórica y forzando, muchas veces, la realidad europea – específicamente la francesa – a la de Latinoamérica. En tal sentido, se advierte entre los objetivos de los autores atender a la complejidad de los procesos institucionales cuestionando "la idea foucaultiana de que la psiquiatría era el brazo biopolítico del Estado para el control de sujetos pobres y marginales" con "conductas que trasgredieran lo que debía considerarse como "normal" (Ríos, 2022, p.547). En el caso de España, Chile o Argentina – por nombrar algunos casos –, la poca capacidad administrativa, el desinterés de los gobiernos, los bajos presupuestos, las precariedades logísticas y materiales fueron el común denominador, en muchos casos quienes abanderaron estas instituciones fungieron más como administradores y recursivos recaudadores de dineros para el sostenimiento de estos establecimientos. Muestra de tal olvido estatal fue la función terapéutica del aislamiento en sí mismo – con gran fama en el periodo decimonónico – como el punto de partida de estos establecimientos. Definitivamente no siempre el hospital para enfermos mentales ejerció un régimen de control sobre los usuarios, y, aún con infraestructuras de imitación panóptica como en el caso cubano, los métodos de vigilancia unidireccional no necesariamente fueron practicados. Y claro que hubo influencia extranjera, pero se trató más de procesos de apropiación local que terminaron por gestar nuevas formas de enfrentar el problema de la locura.

Sobresale a lo largo de los capítulos la pregunta por el "para qué" de estas instituciones, porque una vez consolidadas las edificaciones, fueron continuas las disputas, acuerdos y alianzas entre distintos frentes de participación al tratarse de escenarios híbridos en términos del poder. En cuanto a las iniciativas urbanas destinadas a la construcción de manicomios, la variedad fue la norma; estas podían venir de un frente gubernamental, del sector privado o producto de negociaciones, al tratarse de espacios afines a discusiones filosóficas, médicas, religiosas y políticas de una época que supo esculpir sus ideales de normalidad. Puntualmente, la Casa General de Dementes en Cuba fue utilizada por las fuerzas revolucionarias como bandera de lucha social, primero al ser denunciada por el abandono y las malas condiciones perpetuadas por los gobiernos de ocupación y por el régimen anterior, y, luego, al convertirse en símbolo de transformación social con la victoria del movimiento revolucionario (Lambe, 2022, p.207). En general, las consecuencias de tales condiciones se vieron reflejadas en arquitecturas donde podía encontrarse una botica para el resguardo de fórmulas magistrales contigua a una capilla con altar de mármol y flores de estación para decorar las alabanzas celestiales.

Por último, cabe resaltar el cambio de perspectiva en la mirada histórica, porque usualmente la voz más escuchada ha sido la del saber médico en cabeza de sus protagonistas. Incluso se ha llegado a creer que la historia de estos personajes enaltecidos por sus acciones pioneras es al tiempo un panorama completo que alcanza a narrar la cotidianidad al interior del manicomio. Pero hacer del espacio un personaje central implica entenderlo como el epicentro de interacciones sociales entre distintos actores que vivieron el interior institucional con dinámicas diversas; y es justamente en el contenido de este compilado la posibilidad de escuchar otras fuentes, otras historias que incluyen voces tenues, sutiles o ignoradas. El aporte de esta publicación es la de explorar caminos diversos al ver al manicomio más allá de su pasado oscuro y claro que fue un espacio para la reclusión de la locura, pero también fue el lugar posible de la experimentación terapéutica, de la producción de conocimiento en el campo de la psiquiatría, de la conformación de grupos académicos, de la profesionalización del personal y – en ocasiones – el único refugio para muchos otros.


REFERENCIAS

FRIGERIO Graciela Bosquejos para pensar juntos los oficios del lazo: cuidar, curar, educar 30 10 2022 https://www.youtube.com/watch?v=5zE8vgcf6To&t=43s&ab_channel=TeleducacionFacultaddeMedicinaUniversidaddeAntioquia Consultado el: 30 oct. 2022
LAMBE Jennifer Mazorra, el bedlam de Cuba: de la casa general de dementes al hospital psiquiátrico de La Habana, 1857-1980 Ríos Andrés Ruperthuz Mariano org De manicomios a instituciones psiquiátricas: experiencias en Iberoamérica, siglos XIX y XX Ciudad de México Unam Madrid Sílex Ediciones S.L 2022 541 580
RÍOS Andrés Terapias de choque para ricos en la capital mexicana: la clínica neuropsiquiátrica Samuel Ramírez Moreno, 1931-1951 Ríos Andrés Ruperthuz Mariano org De manicomios a instituciones psiquiátricas: experiencias en Iberoamérica, siglos XIX y XX Ciudad de México Unam Madrid Sílex Ediciones S.L 2022 541 580
RÍOS Andrés RUPERTHUZ Mariano org De manicomios a instituciones psiquiátricas: experiencias en Iberoamérica, siglos XIX y XX Ciudad de México Unam Madrid Sílex Ediciones S.L 2022
